# Exploring diflunisal as a synergistic agent against *Staphylococcus aureus* biofilm formation

**DOI:** 10.3389/fmicb.2024.1399996

**Published:** 2024-09-25

**Authors:** Maria Salazar, Siavash Shahbazi Nia, Nadezhda A. German, Babafela Awosile, Saheed Sabiu, Alexandra Calle

**Affiliations:** ^1^School of Veterinary Medicine, Texas Tech University, Amarillo, TX, United States; ^2^School of Pharmacy, Department of Pharmaceutical Sciences, Texas Tech University Health Sciences Center, Amarillo, TX, United States; ^3^Faculty of Applied Sciences, Department of Biotechnology and Food Science, Durban University of Technology, Durban, South Africa

**Keywords:** *Staphylococcus aureus*, biofilms, antibiotics, NSAID, diflunisal

## Abstract

*Staphylococcus aureus* is a bacterial pathogen of considerable significance in public health, capable of inducing a diverse range of infectious diseases. One of the most notorious mechanisms used by *S. aureus* to survive and colonize the site of infection is its ability to form biofilms. Diflunisal, a non-steroidal anti-inflammatory drug (NSAID), is a known inhibitor of the Agr system in *S. aureus*, which is key in regulating biofilm formation. This study evaluated the effect of broad-spectrum antibiotics in combination with diflunisal on *S. aureus* biofilm density. Eight antibiotics were tested independently at different concentrations and in combination with diflunisal to assess their effect on *S. aureus* biofilm formation. When using the antibiotics alone and with diflunisal, a significant control effect on biofilm formation was observed (*p* < 0.05), irrespective of diflunisal presence, but did not achieve a complete biofilm growth inhibition. Over time, diflunisal influenced biofilm formation; however, such an effect was correlated with antibiotic concentration and exposure time. With amikacin treatments, biofilm density increased with extended exposure time. In the case of imipenem, doripenem, levofloxacin, and ciprofloxacin, lower doses and absence of diflunisal showed higher control over biofilm growth with longer exposure. However, in all cases, diflunisal did not significantly affect the treatment effect on biofilm formation. In the absence of antibiotics, diflunisal significantly reduced biofilm formation by 53.12% (*p* < 0.05). This study suggests that diflunisal could be a potential treatment to control *S. aureus* biofilms, but it does not enhance biofilm inhibition when combined with antibiotics.

## 1 Introduction

*Staphylococcus aureus* is a versatile microorganism that can exist both as a pathogenic and commensal organism. Pathogenic strains, which are mostly coagulase-positive staphylococci (CoPs), are responsible for a variety of infections ranging from mild to severe. These infections include conditions such as dermatitis, folliculitis, staphylococcal scalded skin syndrome, toxic shock syndrome, and staphylococcal foodborne disease (Lisowska-Łysiak et al., 2021). As a commensal organism, *S. aureus* can be found on the skin, mucous membranes, and gastrointestinal tract of healthy humans and animals (Rosenberg et al., 2011; Monaco et al., 2017). However, the carriage of this bacteria has been associated with an increased risk of infections influenced by the mechanisms utilized by *S. aureus* to remain indetectable in the host (Sasson et al., 2017). These mechanisms include virulence factors such as the staphylococcal protein A, fibronectin-binding proteins A and B, clumping factors A and B, collagen-binding protein, the capsule, alpha-toxin, and chemotaxis inhibitory protein are associated with the immune response evasion (de Haas et al., 2004; O’Riordan and Lee, 2004; Monecke et al., 2014; Ghasemian et al., 2015; Foster, 2016), and finally the enterotoxins, toxic shock syndrome-toxin-1, and exfoliative toxins, promote toxin-mediated infections (Piano, 2004; Ortega et al., 2010; Dumitrescu et al., 2011; Oogai et al., 2011; Tuffs et al., 2019; Fanelli et al., 2022).

One of the most remarkable features of *S. aureus* is its ability to form biofilms that attach to surfaces, such as body tissues of animals and humans, food contact surfaces, medical devices, and body implants (Donlan, 2002). In *S. aureus,* biofilm attachment is facilitated by protein A, Fibrinogen-binding proteins (FnBPA and FnBPB), biofilm-associated protein (Bap), clumping factor B, and extracellular adherence protein (Herman-Bausier et al., 2015; Nguyen et al., 2020). The biofilm-forming process is regulated by quorum sensing and the Agr system, which consists of proteins AgrA, AgrB, and AgrC, that produce the autoinducer peptide signal (AIPs), activating signaling cascades that lead to the expression of virulence genes, including biofilm formation (Yarwood and Schlievert, 2003; Preda and Săndulescu, 2019; Zhou et al., 2020). Biofilm formation is a common characteristic in *S. aureus* chronic wounds and implant-associated infections (Roy et al., 2021; Tuon et al., 2023). The National Institutes of Health (NIH) and Centers for Disease Control and Prevention (CDC) reported that biofilm infections account for 80% of the total number of microbial infections worldwide (Sen et al., 2021; Assefa and Amare, 2022; Devanga Ragupathi et al., 2022), and the most common bacteria causing biofilm infections is methicillin-resistant *Staphylococcus aureus* (MRSA) (Piechota et al., 2018; Silva et al., 2021).

*Staphylococcus aureus* has exhibited an outstanding ability to evolve resistance against a wide array of antibiotics, being a pressing challenge, especially within clinical and healthcare environments. *Staphylococcus aureus* demonstrated resistance to penicillin shortly after its introduction as an antibiotic (Chambers and DeLeo, 2009). The subsequent advent of methicillin marked a key moment, as it led to the emergence of MRSA, a critical event considering that this microorganism is one of the most widespread and persistent antibiotic-resistant pathogens on a global scale (Hassoun et al., 2017; Guo et al., 2020). MRSA is a well-known resistant type of *S. aureus* to multiple antibiotic groups, including beta-lactams, fluoroquinolones, and tetracyclines, among others (Kaur and Chate, 2015), which makes it a great threat (Turner et al., 2019). In addition, MRSA has been reported to be present in healthy individuals and livestock (Lee et al., 2018), increasing the risk of developing MRSA infections.

Recent studies have explored the use of broad-spectrum antibiotics commonly used on Gram-negative bacteria as alternatives for Gram-positive resistant strains. Examples of these antibiotics include amikacin, gentamicin, and tobramycin, which have been shown to be highly effective against *S. aureus*, including MRSA strains, due to their time and concentration-dependent properties (Hess et al., 2011; Ghazi et al., 2017; Waryah et al., 2017; Broussou et al., 2018; Higashihira et al., 2022; Chaves and Tadi, 2023). Other antibiotics, such as levofloxacin, ciprofloxacin, imipenem, meropenem, and doripenem, have antimicrobial activity against *S. aureus*. When administered in combination with other antibiotics, enhanced activity has been observed, even on *S. aureus* biofilms (Ribes et al., 2010; Amin et al., 2015; Ferreira et al., 2017; Yasir et al., 2021; Podder and Sadiq, 2023; Yee et al., 2022).

On the other hand, NSAIDs have been proposed as a treatment for microbial infections (McCloskey et al., 2016; Carey et al., 2020). Some of these drugs have been proven to have antimicrobial and antibiofilm activity, such as acetylsalicylic acid, aspirin, ibuprofen, celecoxib, and diflunisal (Chan et al., 2017; Öztürk et al., 2021; Paes Leme and da silva, 2021). Diflunisal is a known NSAID approved by the Food and Drug Administration (FDA). It is an inhibitor of the *S. aureus* Agr system, which blocks the expression of virulence factors, including biofilm formation and pathogenesis in general (Hendrix et al., 2016).

Considering the potential of diflunisal to inhibit biofilms, this investigation evaluated the effect of this NSAID on *S. aureus* biofilm formation when combined with antibiotics. We explored whether a synergistic effect exists between the antibiotics and diflunisal to influence *S. aureus* biofilm density.

## 2 Materials and methods

### 2.1 *Staphylococcus aureus* isolates preparation

Five *S. aureus* isolates from the strains library at the Applied Microbiology and Food Safety Laboratory, School of Veterinary Medicine, Texas Tech University, were used. These strains were selected after testing a larger group of isolates based on their ability to form biofilm at similar rates. A drug-resistant and biofilm-forming *Staphylococcus aureus* subsp. *aureus* Rosenbach ATCC 43300 was included as a reference strain/positive control. The isolates were cryopreserved at −80°C with 20% glycerol, and prior to the experiments, they were streaked onto Tryptic Soy Agar (TSA) (Remel, Lenexa, KS, United States) and incubated at 37°C for 24 h. After incubation time, one colony was transferred to a test tube containing 9 mL Brain Heart Infusion broth (BHI) (Millipore Sigma, Burlington, MA, United States) and incubated in motion at 37°C for 24 h at 200 rpm. Upon incubation, a bacterial suspension was created by transferring 0.1 mL of the overnight BHI culture into a 9.9 mL BHI tube and mixing thoroughly to obtain a starting optical density (OD) of 0.003 ± 0.001 at OD 600 nm (approximately 10^7^ CFU/mL), which was measured by transferring 100 μL of the bacterial suspension into a 96-well plate and using a microplate reader. Strains were grown and tested individually.

### 2.2 Biofilm formation and quantification using the crystal violet staining method

To allow biofilm formation, we used the microtiter dish biofilm formation assay described elsewhere (O'Toole, 2011). In brief, from the bacterial suspension prepared for each strain, 200 μL were deposited in triplicate into a 96-flat bottom well plate (Costar®, Washington, D.C., United States) and incubated at 37°C for 48 h in static conditions to permit biofilm formation. Upon incubation, the broth was removed from each well, and the wells were rinsed three times with 300 μL of sterile distilled water. To aid the biofilms being fixed in the wells, 200 μL of 90% ethanol were added and left for 15 min. Finally, the ethanol was removed, and the plates were allowed to air dry for 1 h. The biofilm was stained with 200 μL of 1% crystal violet (CV) (Carolina®, Burlington, NC, United States), which remained in the wells for 45 min. The CV was removed, and the 96-well plate was washed by submerging it in distilled water. The latter was repeated three times. Lastly, upside-down plates were allowed to air dry overnight at room temperature, and biofilms were formed at the bottom of each well. Biofilm density was quantified further by measuring optical density. A 200 μL aliquot of 70% ethanol was added to each well to destain the CV and allowed to set for 3 min. Following this, 100 μL of the last suspension was transferred to a new 96-well plate. Subsequently, the plate was placed on a Microplate Reader LX Multi-Mode Microplate Reader (Synergy LX Multi-Mode, BioTek, Winooski, VT, United States) to measure absorbance at optical density (OD) of 580 nm.

### 2.3 Preparation of antibiotics and diflunisal

Antibiotics were selected based on mode of action, concentration, and exposure time. The antibiotics tested were grouped based on the key aspect of their effectiveness: concentration-dependent antibiotics (amikacin, tobramycin, gentamicin, levofloxacin, and ciprofloxacin), and time-dependent antibiotics (imipenem, meropenem, and doripenem). The inclusion of both types of antibiotic activities for the experimental design allows for a thorough evaluation of biofilm formation under different antibiotic exposure dynamics. Diflunisal was chosen due to its documented inhibitory effects on the disruption of *S. aureus* Agr system. All chemical compounds were provided by the Department of Pharmaceutical Sciences, School of Pharmacy, Texas Tech University Health Sciences Center, in Amarillo, Texas. Based on their solubility, antibiotics and diflunisal were reconstituted in autoclaved distilled water (DW) or dimethyl sulfoxide (DMSO) (Sigma-Aldrich, St. Louis, MO, United States) (Table 1). After reconstitution, the stock solutions of all compounds were stored at −20°C. For each experiment, stock solutions were thawed in a water bath at room temperature and diluted in autoclaved DW to obtain the desired concentrations. The antibiotics were tested at different concentrations, and the concentrations chosen were based on the quality control range of acceptable minimum inhibitory concentration (MIC) for *S. aureus* provided by the M100 Performance Standards for Susceptibility Testing document by Clinical and Laboratory Standards Institute (2021). Diflunisal was tested only at one concentration (25 μg/mL), and this was chosen based on literature in which it is reported that at this dose, it inhibits *S. aureus* quorum sensing (Bernabè et al., 2021). Table 1 describes the details and specific concentrations of each compound tested.

**Table 1:** Antibiotics and diflunisal information.

*Drug*	*Compound*	*Molecular weight*	*Class*	*Spectrum*	*Concentrations tested (μg/mL)*	*Solvent*
Amikacin	Amikacin sulfate	781.76	Aminoglycoside	Broad	1.0 and 4.0	Water
Tobramycin	Tobramycin sulfate	565.59	Aminoglycoside	Broad	0.12 and 1.0	Water
Gentamicin	Gentamicin sulfate	575.67	Aminoglycoside	Broad	0.12 and 1.0	Water
Imipenem	Imipenem monohydrate	317.36	Carbapenem	Broad	0.016 and 0.06	DMSO
Meropenem	Meropenem trihydrate	437.51	Carbapenem	Broad	0.03 and 0.12	Water
Doripenem	Doripenem monohydrate	438.52	Carbapenem	Broad	0.016 and 0.06	DMSO
Levofloxacin	Levofloxacin	740.75	Quinolone	Broad	0.06 and 0.5	DMSO
Ciprofloxacin	Ciprofloxacin	331.34	Fluoroquinolones	Broad	0.12, 0.5, and 2.0	DMSO
Diflunisal	Diflunisal	250.2	NSAID	Not applicable	25	DMSO

^1^Concentrations according to the M100 Performance Standards for Susceptibility Testing document by Clinical and Laboratory Standards Institute (2021).

### 2.4 Concentration effect of antibiotics and diflunisal on *Staphylococcus aureus* biofilm formation

For this set of experiments, a total of eight antibiotics were tested with and without diflunisal. Two different treatment scenarios were investigated. The first scenario was the individual treatment, which consisted of testing diflunisal individually (no antibiotic) and each antibiotic independently without diflunisal on one *S. aureus* strain at the time by adding into 100 μL of the target drug into a well of a 96-well plate, followed by 100 μL of bacterial suspension. The second scenario consisted of a co-treatment, which involved the evaluation of the antibiotics in combination with diflunisal on each *S. aureus* strain; in this case, 50 μL of diflunisal solution, 50 μL of the antibiotic solution, and 100 μL of the bacterial suspension were added into a well. In both cases, experiments were conducted in triplicate; plates were incubated at 37°C for 48 h under static conditions to allow biofilm formation. Upon incubation, biofilm density (OD 580 nm) was quantified using crystal violet as described in section 2.2. Specific concentrations of the antibiotics and diflunisal are listed in Table 1.

### 2.5 Time effect on *Staphylococcus aureus* biofilm density treated with antibiotics and diflunisal

For these experiments, only amikacin, imipenem, doripenem, levofloxacin, and ciprofloxacin were tested. The selection of these five antibiotics was based on results from the previous experiments, as they exhibited the highest effect in controlling *S. aureus* biofilm formation. To evaluate the effect of antibiotics in combination with diflunisal over time, each isolate was subjected to biofilm growth individually and treated with the five antibiotics, both in the presence and absence of diflunisal. Biofilm density was measured every 4 h at an OD of 580 nm, during 48 h. The selection of time points was considered based on the different stages of growth and biofilm formation.

### 2.6 *Staphylococcus aureus* planktonic cells growth with antibiotics and diflunisal

To evaluate the effect of diflunisal in combination with antibiotics over time on *S. aureus* planktonic growth, growth curves for the isolates used were created by incubating each isolate independently in BHI broth at 37°C with each of the five antibiotics previously chosen, using the same concentrations tested for biofilm formation as described in Table 1. Experiments were conducted with and without diflunisal. The treatments of antibiotics without diflunisal were conducted to evaluate if there was a statistically significant difference between the effect of antibiotics alone and combined with diflunisal on planktonic growth, while untreated *S. aureus* served as the control to establish a baseline for comparison. Bacterial concentration was measured over time every 4 h for 48 h based on absorbance at an OD of 600 nm. To analyze the data, the absorbance (OD 600 nm) values obtained for each isolate (including *S. aureus* ATCC 43300) were averaged.

### 2.7 LCMS/MS quantification

Antibiotics were quantified using a combination of previously reported methods with modifications. Briefly, 10 mg/mL stock solutions of the test drugs were prepared in either water or DMSO. After serial dilutions, 25 μg/mL solution of diflunisal (in BHI) was mixed with 25 μg/mL solution of each antibiotic (in BHI). The standard curve for diflunisal was also prepared in the absence of antibiotics to accurately quantify the concentration of diflunisal without interference from antibiotics. From each sample, test sample, or standard curve sample, new samples were prepared composed of 50 μL of diflunisal-antibiotic sample or standard curve sample + 50 μL of internal standard (prepared in ACN: H2O, 70: 30) + 150 μL of ACN: H2O, 70: 30. The samples were quantified using LCMS/MS via the following method: For analysis of samples, AB SCIEX QTRAP 5500 triple quadrupole mass spectrometer, attached to a Nexera UPLC system (Shimadzu Corporation), was used. The UPLC system contained an autosampler (Sil-30 AC), pumps (LC-30 AD), a controller (CBM-20A), a degasser (DGA-20A5), and a column oven (CTO-30A). Analyst software was used for data acquisition and quantification. Detailed information for the chromatographic separation of test compounds is summarized below. Column: Gemini-C18 (2 mm × 50 mm, 3 μm; Phenomenex, Torrance, CA, United States); mobile phases: (A): 2 mM Ammonium Formate in water, (B): ACN; flow rate: 0.4 mL/min; gradient: 0–0:10 min; 30% B, 0:10–1 min; to 90% B, 1:0–2:0 min; 90% B, 2:0–2:10 min; to 30% B, 2:10–3:0 min; 30% B.

### 2.8 *Staphylococcus aureus* biofilm viability and microscopy assay

A microscopy live/dead assay of *S. aureus* cells within the biofilms subjected to the treatments was performed. Biofilms were grown and treated according to the protocols described in sections 2.2 and 2.4, with the assay scaled to a 24-well plate (Corning™ Costar™, Corning, NY) format. Since no statistical differences were observed in biofilm densities across the isolates used, a bacterial mix was prepared by combining equal volumes of the six isolates. From this bacterial cocktail, a suspension was created by diluting 0.1 mL of the cocktail in 9.9 mL of BHI. To treat the biofilms with antibiotics individually, 500 μL of the drug was added to each well, followed by 500 μL of the bacterial suspension, resulting in a final volume of 1 mL. For treatments combining antibiotics with diflunisal, 250 μL of the NSAID, 250 μL of the antibiotic, and 500 μL of the bacterial suspension were added to each well. The plate was incubated as specified above. After incubation, the remaining media was removed, and the wells were washed three times with sterile distilled water. Biofilms were prepared for microscopy using the Live/Dead BacLight Bacterial Viability Kit (Molecular Probes, Inc., Eugene, Oregon). A staining solution was prepared by mixing 1 μL of Propidium iodide (for dead cells) and Syto 9 (for live cells) with 148 μL of sterile distilled water per well. The solution was added to each well, and the plate was incubated at room temperature for 15 min, protected from light. Subsequently, confocal laser scanning microscopy (CLSM) was performed using the Leica Stellaris 8 Falcon STED Confocal Microscope (Leica Microsystems, Wetzlar, Germany) to visualize the cells in the biofilms.

### 2.9 Statistical analysis

Data management was done using a Microsoft Excel spreadsheet (2016, Microsoft Corp., Redmond, WA), and it was imported into R software for analysis. For the evaluation of the effect of antibiotics and diflunisal on *S. aureus* biofilms after 48 h of treatment, the focus of the analysis was to explore if there was any statistical difference in *S. aureus* biofilm density (OD 580 nm) between each treatment type (i.e., antibiotics versus antibiotics combined with diflunisal, at the different antibiotics concentrations). Initial descriptive statistics were carried out to summarize *S. aureus* biofilm density after the treatments and were presented graphically as a mean plot with respective standard error of the mean. For data analysis, a two-way ANOVA was used. Considering the eight antibiotics (amikacin, tobramycin, gentamicin, imipenem, meropenem, doripenem, levofloxacin, and ciprofloxacin) with different concentrations; eight different two-way ANOVA were conducted. In each model, standard parametric model assumptions for two-way ANOVA were explored. The Shapiro–Wilk test and quantile plot were used to explore the normality test statistically and graphically, respectively. The assumption of homoscedasticity was employed using Levene’s test. Whenever there was a violation of parametric assumption, robust two-way ANOVA was conducted. When a variable was statistically significant, a *post-hoc* comparison of the treatment type and/or antimicrobial concentrations was carried out, adjusting for multiple comparisons using Bonferroni correction.

On the other hand, to analyze the effect of the five antibiotics chosen (amikacin, imipenem, doripenem, levofloxacin, and ciprofloxacin) with or without diflunisal in biofilm density and planktonic growth over time (at 4 h intervals for 48 h), we focused our analysis in exploring the difference in *S. aureus* biofilm and planktonic growth densities over time with the different antibiotic concentrations, individually or in combination with diflunisal. Initial descriptive statistics were carried out to summarize outcomes of interest over time and were presented graphically as mean plots with respective standard error of mean. For data analysis, a repeated measured ANOVA was used to account for possible correlation in *S. aureus* biofilm and planktonic growth densities over time. For each antibiotic, a repeated measured ANOVA was performed for all the *S. aureus* biofilm and planktonic growth densities as outcome variables. In each model, standard parametric model assumptions for repeated measure ANOVA were explored. Shapiro–Wilk test and quantile plot were used to explore the normality test statistically and graphically, respectively. Possible outlying observations were also investigated. The assumption of sphericity was explored using Mauchly’s test; whenever there was a violation of the sphericity assumption, Greenhouse–Geisser sphericity correction was applied to factors violating this assumption. Whenever there is a statistically significant interaction effect between the antibiotic concentration and time factors in the model, a statistical comparison of the concentration at each time point was done, adjusting for multiple comparisons, using Bonferroni correction. The interaction effects were presented graphically to explain the effects of these factors on the *S. aureus* biofilm and planktonic densities.

A statistically significant difference between the factors was considered at *p* < 0.05. All analyses were done using the computing environment of R software (v 4.2.2) using the appropriate packages.

## 3 Results

### 3.1 Biofilm density quantification

**Figure 1:**
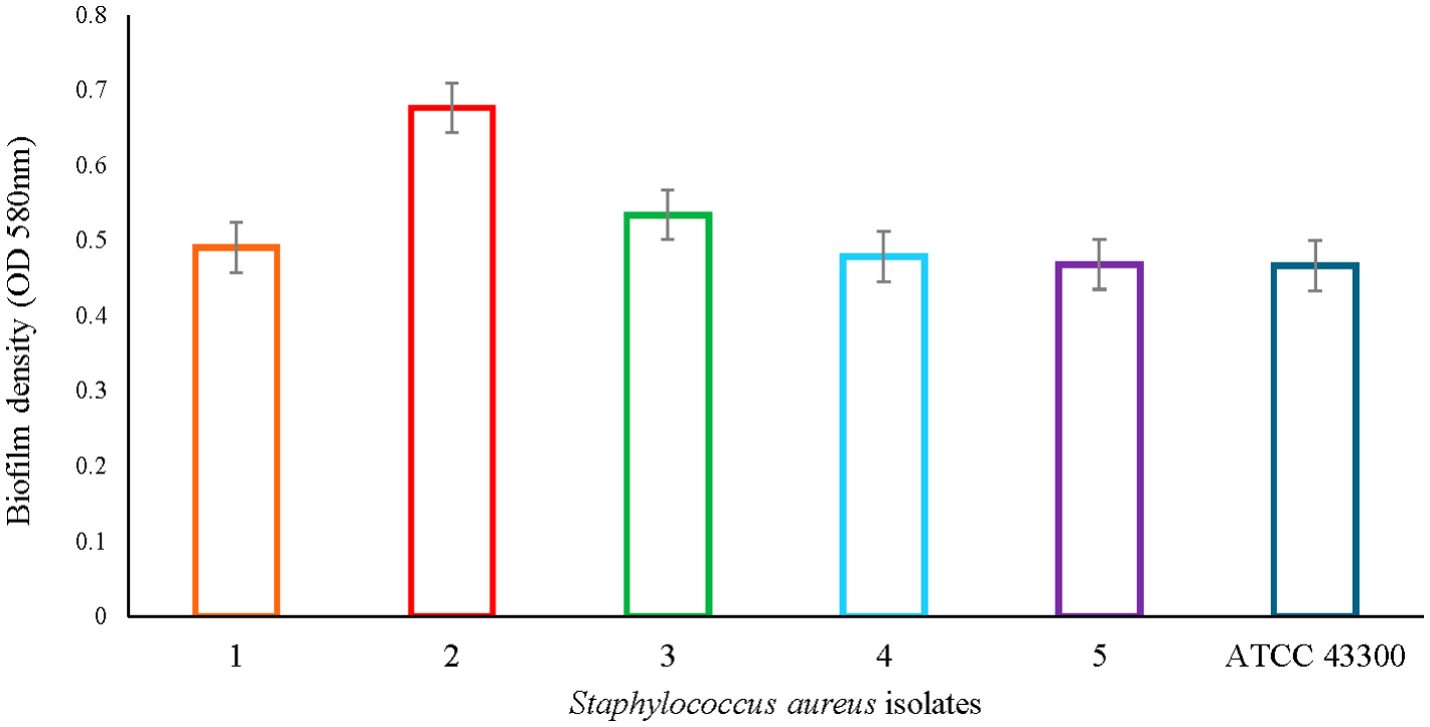
*Staphylococcus aureus* biofilm density. Colored bars represent OD values based on absorbance (OD 580 nm) ± standard error of mean (SEM) per each S. aureus isolate. No statistical difference was observed between all six strains.

*Staphylococcus aureus* subsp. *aureus* Rosenbach ATCC 43300 (known biofilm producer) was used as a reference strain for biofilm formation. After 48 h of incubation, its biofilm density average was 0.466 ± 0.04 (OD 580 nm) (Figure 1), and this value was used to determine biofilm production by *S. aureus* strains selected for this study. As depicted in Figure 1, all isolates evaluated were biofilm producers. The OD values for biofilm density were 0.49 ± 0.06, 0.67 ± 0.07, 0.53 ± 0.08, 0.48 ± 0.05, and 0.47 ± 0.05, for isolates 1 through 5, respectively. No statistical difference between the strains was observed (*p* > 0.05). The OD average for all isolates was 0.529 ± 0.02, and this value was used as the point of reference or baseline for the data analysis pertaining to the antibiotics and diflunisal effect on biofilm formation.

### 3.2 Effect of antibiotics concentration and diflunisal on biofilm density

**Figure 2:**
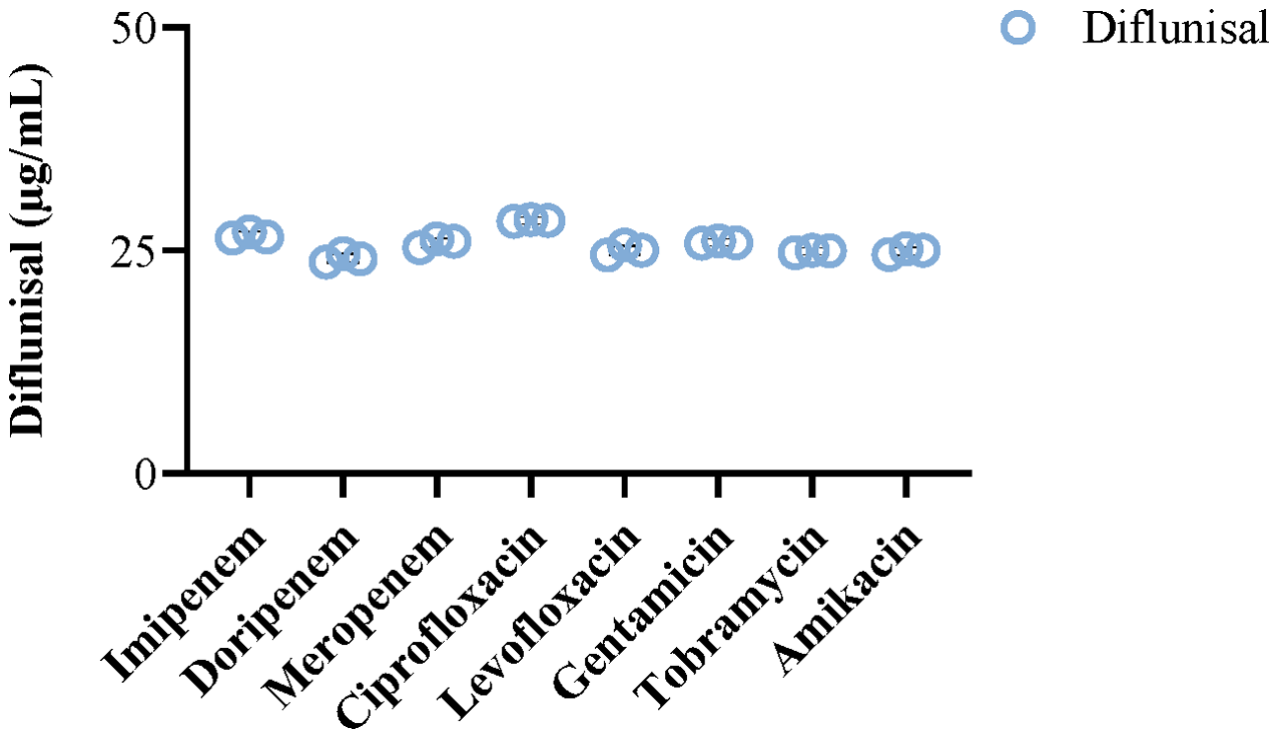
Impact of selected antibiotics on the solubility of diflunisal. The addition of various antibiotics (25 μg/mL) did not alter the solubility of diflunisal and its final concentration (25 μg/mL) in BHI media. The bar histograms represent mean ± SEM (*N* = 3).

Changes in biofilm density were monitored in the presence of antibiotics with and without diflunisal. Considering that the chemical structures of the utilized antibiotics contain amine groups and given the inherently acidic nature of diflunisal (pKa 2.9), we evaluated if alterations in pH caused by mixing of various antibiotics with diflunisal could change the solubility of compounds in the media. Results showed no noticeable alteration in the solubility of the antibiotics when mixed with diflunisal vs. individual samples (Figure 2), ensuring the accurate delivery of proposed drug concentrations in the following experiments.

Biofilm density was tested after the antibiotic treatments with and without diflunisal. After the growth period of 48 h, statistical analysis comparing density before and after treatment revealed a reduction in biofilm formation, which was influenced by the antibiotic dose (*p* < 0.01) irrespective of diflunisal. However, when we compared the level of reductions achieved between treatments with diflunisal and without diflunisal, no significant differences (*p* > 0.05) were observed; in other words, the presence of diflunisal in combination with the antibiotics did not influence biofilm density. Table 2 describes the antibiotics tested, the concentrations, the percentage of biofilm reduction per each treatment, and their corresponding *p* value.

**Table 2:** Biofilm density changes when treated with antibiotics in the presence and absence of diflunisal.

Antibiotic	Inhibition of biofilm formation^1^	
	Concentration of AB dose without diflunisal^2^	Concentration of AB dose with diflunisal^2^
Amikacin	1 μg/mL = 53.88%	1 μg/mL = 39.32%
	4 μg/mL = 64.46%	4 μg/mL = 62.76%
Tobramycin	0.12 μg/mL = 41.21%	1 μg/mL = 41.02%
	1 μg/mL = 27.98%	4 μg/mL = 33.65%
Gentamicin	0.12 μg/mL = 42.72%	0.12 μg/mL = 38.37%
	1 μg/mL = 48.58%	1 μg/mL = 50.66%
Imipenem	0.016 μg/mL = 51.04%	0.016 μg/mL = 36.48%
	0.06 μg/mL = 45.56%	0.06 μg/mL = 31.38%
Meropenem	0.03 μg/mL = 42.16%	0.03 μg/mL = 21.74%
	0.12 μg/mL = 43.48%	0.12 μg/mL = 31.76%
Doripenem	0.016 μg/mL = 68.06%	0.016 μg/mL = 55.01%
	0.06 μg/mL = 76.94%	0.06 μg/mL = 75.24%
Levofloxacin	0.06 μg/mL = 40.64%	0.06 μg/mL = 32.51%
	0.5 μg/mL = 76.56%	0.5 μg/mL = 72.02%
Ciprofloxacin	0.12 μg/mL 48.58%	0.12 μg/mL = 32.51%
	0.5 μg/mL = 68.62%	0.5 μg/mL = 76.37%
	2 μg/mL = 74.67%	2 μg/mL = 73.53%

1 Percentage of inhibition based on biofilm density reduction; results are comparing control and treatment.

2 Values expressed in μg/mL indicate the concentration of the antibiotic tested. Values in percentage indicate the reduction in biofilm formation with respect to the control.

The effect of diflunisal alone (in the absence of any antibiotics) on *S. aureus* biofilm was tested using a concentration of 25 μg/mL. Findings show at the end of the growth period (48 h), a significant effect (*p* < 0.0001) in reducing biofilm density by 53.12%.

### 3.3 Effect of antibiotics and diflunisal on *Staphylococcus aureus* biofilm over time

Biofilm density was clearly affected by exposure time. In the case of amikacin treatments, a prolonged time of exposure led to an increase in biofilm density. Conversely, fluctuations in biofilm formation over time were observed at specific intervals with imipenem, doripenem, levofloxacin, and ciprofloxacin. In general, at the lower doses of the antibiotics and in the absence of diflunisal, longer exposure demonstrated higher control over biofilm growth. Additionally, the time-dependent effect of antibiotic concentration on biofilm formation was evident in all treatments.

**Figure 3:**
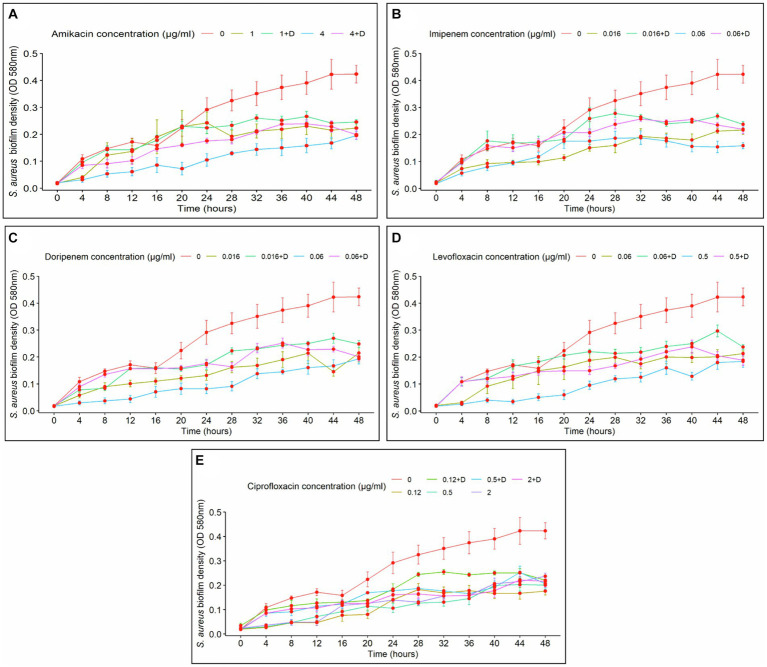
*Staphylococcus aureus* biofilm density over time, depending on the concentration of the antibiotics with (+D) or without diflunisal. **(A)** Amikacin, **(B)** Imipenem, **(C)** Doripenem, **(D)** Levofloxacin, **(E)** Ciprofloxacin. In most cases, antibiotic concentration had a time-dependent impact on biofilm formation. Data are represented as the average of the *S. aureus* isolates biofilm density (OD 580 nm) ± standard error of mean (SEM) every 4 h, during 48 h of incubation. The repeated measure ANOVA was significant at *p* < 0.0001.

The effects of amikacin concentration (with and without diflunisal) and time on biofilm density were significant (*p* < 0.0001). As shown in Figure 3A, at 1 μg/mL and 4 μg/mL of amikacin without diflunisal, a biofilm density increase was observed from 0 to 24 h, but compared to the control group, the biofilm density produced was less. However, when diflunisal was added, a decreased efficacy of the treatment was observed between 28 and 48 h. No statistical difference in biofilm density was observed between treatments. Nonetheless, significant differences were observed among the different concentrations.

Regarding imipenem, significant effects of the antibiotic concentration and time on biofilm density (*p* < 0.0001) were observed, indicating that the impact of the antibiotic concentration was dependent upon the duration of exposure. Biofilm density of *S. aureus* increased over time with both antibiotic concentrations used individually and with diflunisal (Figure 3B). At 0.06 μg/mL of imipenem alone, the antibiotic caused a bacteriostatic effect from the 28-h time point. However, at both 0.016 μg/mL and 0.06 μg/mL concentrations of the antibiotic, the presence of diflunisal resulted in a diminished effect on the treatment over time. Nonetheless, there were no significant differences between the treatments in *S. aureus* biofilm density at 0.016 μg/mL and 0.06 μg/mL (except at 4 and 8 h time points, *p* < 0.036). Significant differences were observed among the control group, 0.016 μg/mL, and 0.06 μg/mL imipenem concentrations, regardless of diflunisal presence.

In the case of doripenem (Figure 3C), the ANOVA demonstrated that both doripenem concentrations and time had statistically significant effects on biofilm density (*p* < 0.0001). Biofilm density increased over time with 0.016 μg/mL and 0.06 μg/mL of the antibiotic alone and in combination with diflunisal. Additionally, at both concentrations of the antibiotic, the presence of diflunisal promoted biofilm growth, compared to the effect of the antibiotic alone. However, there were no statistically significant differences in *S. aureus* biofilm density between the treatments, except with 0.016 μg/mL at 44 h (*p* = 0.018), and 0.06 μg/mL at 4, 8, 12, and 36 h (*p* < 0.007). In addition, significant differences in *S. aureus* biofilm density were observed among the control group, 0.016 μg/mL, and 0.06 μg/mL concentrations of doripenem, regardless of diflunisal presence.

Levofloxacin effect on *S. aureus* biofilm formation is represented in Figure 3D. The repeated measure analysis of variance (ANOVA) indicated that both levofloxacin concentrations and time had statistically significant effects on biofilm density (*p* < 0.0001). Biofilm density increased over time in presence of both concentrations of levofloxacin with and without diflunisal. However, with 0.5 μg/mL of levofloxacin in the absence of diflunisal, the biofilm growth was lower compared to the 0.06 μg/mL treatments. Additionally, at both concentrations of the antibiotic, the presence of diflunisal caused a decrease in the treatment efficacy on the growth control of *S. aureus* biofilm over time. However, there were no significant differences in biofilm density with both concentrations of levofloxacin alone and with diflunisal at each time point, except with 0.5 μg/mL at the 20 and 40 h time points, where the biofilm density is higher compared to the treatment without diflunisal. However, there were statistically significant differences in *S. aureus* biofilm density among the control group, 0.06 μg/mL, and 0.5 μg/mL treatments, regardless of the presence of the NSAID.

Regarding ciprofloxacin, a statistically significant effects of the antibiotic concentration and time on biofilm density (*p* < 0.0001) was observed. As shown in Figure 3E, *S. aureus* biofilm density increased over time irrespective of ciprofloxacin concentrations and the presence of diflunisal. However, the treatment that exerted the most substantial control over biofilm growth was 0.5 μg/mL of ciprofloxacin in the absence of diflunisal between 20 and 36 h. With the three concentrations (0.12 μg/mL, 0.5 μg/mL, and 2 μg/mL), the presence of diflunisal decreased the efficacy of the treatments in controlling biofilm formation over time. However, there were no statistically significant differences in biofilm density observed between the 0.12 μg/mL treatments, the 0.5 μg/mL treatments (except at 28 h, where *p* = 0.041), and the 2 μg/mL treatments at each of the time points. Although, there were some statistically significant differences in *S. aureus* biofilm density between the control group and the treatments.

### 3.4 Effect of diflunisal on *Staphylococcus aureus* biofilm over time

**Figure 4:**
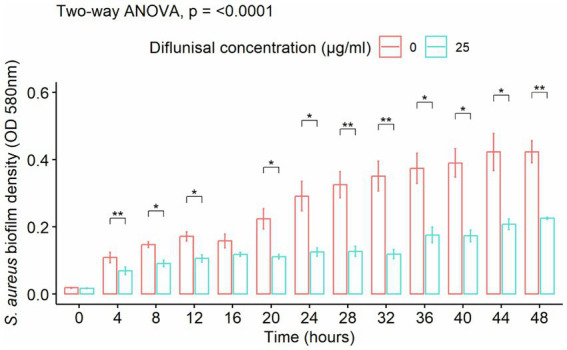
*Staphylococcus aureus* biofilm density over time, treated with 25 μg/mL of diflunisal. Overall, there was an increase in biofilm density over time. However, when comparing the control and treatment, diflunisal controlled biofilm formation over time. Data are represented as the average of S. aureus isolates biofilm density (580 nm) ± standard error of mean (SEM) over time depending on the diflunisal (25 µg/mL) concentration. The repeated measure ANOVA was significant at *p* < 0.0001. Asterisk (*) shows statistically significant difference (*p* < 0.05) between the control group and diflunisal treatment for each time point. Comparison between the groups at each time point was adjusted using Bonferroni correction for multiple comparison.

*Staphylococcus aureus* biofilm was treated with diflunisal alone. In the presence of 25 μg/mL of the NSAID, biofilm formation increased between 0 and 16 h, was static between 16 and 32 h, and increased again after 32 h. From the repeated measure ANOVA, the main effects of diflunisal concentration and time were statistically significant (*p* < 0.001), and the effect of diflunisal concentration on the biofilm density was time-dependent. No significant differences (*p* < 0.05) were observed between control and treatment group during the first 4 h of treatment, however, changes became statistically different comparing control and treatment group after 4 h of diflunisal exposure (Figure 4).

### 3.5 Effect of antibiotics and diflunisal on *Staphylococcus aureus* planktonic growth

**Figure 5:**
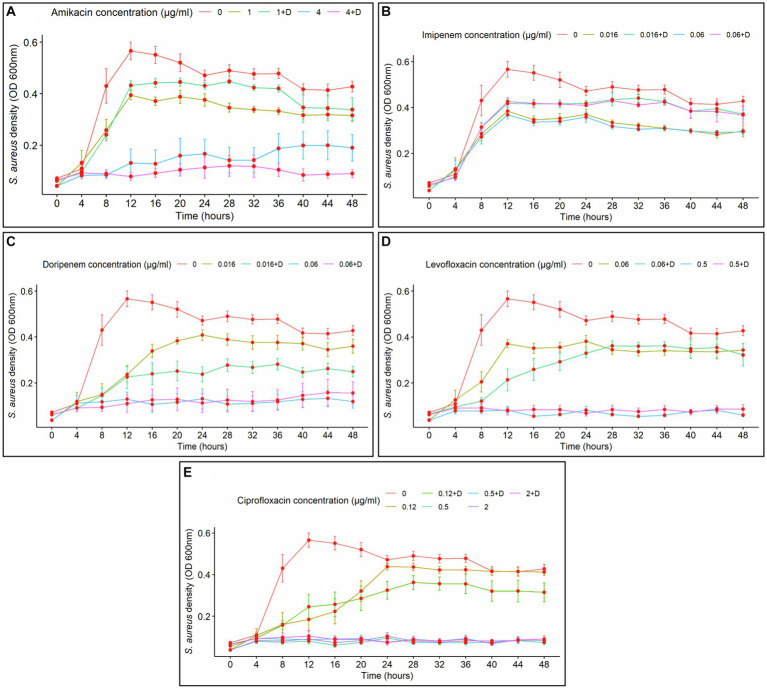
*Staphylococcus aureus* planktonic cell density over time, treated with antibiotics alone and in combination with diflunisal. **(A)** Amikacin, **(B)** Imipenem, **(C)** Doripenem, **(D)** Levofloxacin, **(E)** Ciprofloxacin. Overall, all antibiotics exhibited a bacteriostatic effect on *S. aureus* planktonic growth over time, with different impacts depending on the concentration and the presence of diflunisal. Data are represented as the average of the *S. aureus* isolates planktonic cell density (OD 600 nm) ± standard error of mean (SEM), every 4 h during 48 h of incubation with different concentrations of antibiotics with (+D) and without diflunisal. The repeated measure ANOVA was significant (*p* < 0.0001).

Growth curve assays for *S. aureus* permitted the examination of the impact of five specific antibiotics (amikacin, imipenem, doripenem, levofloxacin, and ciprofloxacin). The average density of untreated *S. aureus* increased over time with an observed lag phase from 0 to 4 h, an exponential phase from 4 to 12 h, and a stationary phase between 12 and 48 h of incubation (Figure 5). Overall, for all treatments, no bacterial reduction or death phase was observed, suggesting that all tested antibiotics produced a bacteriostatic effect.

In the case of amikacin (Figure 5A), with 1 μg/mL, a lag phase was observed from 0 to 4 h, the exponential growth of planktonic cells was observed between 4 and 12 h, and from 12 to 48 h the antibiotic caused a bacteriostatic effect. However, when diflunisal was added, caused a decrease in effectiveness to control bacterial growth control compared to amikacin alone during the 12–48-h period. At 4 μg/mL of amikacin alone and with diflunisal, the treatments caused a bacteriostatic effect, maintained over time. There was a notable difference between the use of 1 μg/mL and 4 μg/mL of amikacin, as the higher concentration suppressed planktonic growth from 4 to 48 h, which explains that the effect of the antibiotic on planktonic cell growth was dose-dependent. Additionally, a two-way interaction was observed between the concentration of amikacin and the exposure time of the bacteria to the antibiotic, indicating that the impact of amikacin concentration on *S. aureus* growth was contingent on time. Except for the 28 and 32-h time points, there was no significant difference in *S. aureus* growth between the use of 1 μg/mL of amikacin alone and in combination with diflunisal, or between the use of 4 μg/mL of amikacin with or without diflunisal. Nonetheless, statistically significant differences in *S. aureus* density were found between the control group (no treatment) and 1 μg/mL or 4 μg/mL of amikacin with or without diflunisal.

Regarding imipenem treatments (Figure 5B), with both concentrations of the antibiotic irrespective of diflunisal presence, exponential growth was observed from 0 to 12 h, and then from 12 to 48 h, the treatments showed a bacteriostatic effect. The statistical analysis indicated that both imipenem concentration (with and without diflunisal) and time, resulted in significant effects on microbial growth (*p* < 0.0001). Moreover, a two-way interaction between imipenem concentration and time was observed, implying that the impact of imipenem concentrations on *S. aureus* growth was dependent upon the duration of exposure to the antibiotic. Except for the 24 h time point, there were no significant differences in *S. aureus* growth between the use of 0.016 μg/mL of imipenem with and without diflunisal at each time point. Likewise, no significant effect was found between the treatment with and without diflunisal for the 0.06 μg/mL imipenem concentration at the different time points. Nevertheless, there were several statistically significant distinctions in *S. aureus* density among the control group and 0.016 μg/mL or 0.06 μg/mL of imipenem with or without diflunisal.

With doripenem treatments (Figure 5C), a bacteriostatic effect maintained over time was observed when using 0.06 μg/mL of the antibiotic individually and in combination with diflunisal, in comparison to 0.016 μg/mL of doripenem with and without the NSAID. At 0.016 μg/mL of doripenem alone, the exponential growth of planktonic cells was observed between 8 and 24 h of treatment, and then from 24 to 48 h the antibiotic controlled bacterial growth. However, the control over bacterial growth was more pronounced when utilizing 0.016 μg/mL of doripenem in combination with diflunisal. Furthermore, the ANOVA revealed that the effects of doripenem concentration (individually and in combination with diflunisal) were statistically significant (*p* < 0.0001), and a two-way interaction was observed between doripenem concentration and time concerning the impact on *S. aureus* growth. Additionally, there were no significant differences in bacterial growth between the use of 0.016 μg/mL of the antibiotic alone and in combination with diflunisal at each time point, except for the 24 h time point. Similarly, there were no statistically significant differences in the effect on *S. aureus* growth between the use of 0.06 μg/mL of doripenem with and without diflunisal at the various time points. Nonetheless, several statistically significant differences in *S. aureus* growth were observed among the control group, as well as the 0.016 μg/mL and 0.06 μg/mL doripenem concentrations with and without the NSAID.

Regarding levofloxacin (Figure 5D), at a concentration of 0.06 μg/mL of the antibiotic alone, *S. aureus* planktonic cell density increased between 0 and 12 h time points, then from 12 to 48 h the antibiotic exerted control over bacterial growth. When using 0.06 μg/mL of levofloxacin in combination with diflunisal, bacterial density increased between 4 and 28 h, and from 28 h to 48 h the mix controlled bacterial growth, maintaining it static. Additionally, when using 0.5 μg/mL of the antibiotic irrespective of diflunisal status, a bacteriostatic effect of the treatments was observed. The ANOVA indicated that both levofloxacin concentrations (individually and in combination with diflunisal) and time caused statistically significant effects on *S. aureus* growth (*p* < 0.0001). Additionally, a two-way interaction was observed between levofloxacin concentration and time in relation to the impact on *S. aureus* growth. There were no statistically significant differences in bacterial growth between the use of 0.06 μg/mL of levofloxacin alone and in combination with diflunisal, as well as between the use of 0.5 μg/mL of the antibiotic with and without the NSAID, at each of the time points. However, there were significant differences in *S. aureus* growth among the control group, the 0.06 μg/mL, and the 0.5 μg/mL levofloxacin concentrations, both individually and with diflunisal.

With respect to ciprofloxacin treatments (Figure 5E) on *S. aureus* growth, the treatments exerted control over bacterial growth in a dose-dependent manner. A bacteriostatic effect of ciprofloxacin was observed from 4 to 48 h at concentrations of 0.5 μg/mL and 2 μg/mL, regardless of the presence of diflunisal, compared to the 0.12 μg/mL treatments. At the 0.12 μg/mL concentration of ciprofloxacin in combination with diflunisal, a decrease in the bacteriostatic effect over *S. aureus* planktonic cells between 12 and 16 h, whereas the growth control was greater between 24 and 48 h compared to the effect of the antibiotic alone. The statistical analysis (ANOVA) revealed significant effects of ciprofloxacin concentration (with and without the NSAID) and time on *S. aureus* growth (*p* < 0.0001), indicating that the impact of antibiotic concentration on bacterial growth was dependent on the duration of exposure. Moreover, there were no statistically significant differences in *S. aureus* planktonic cell growth between the use of 0.12 μg/mL of ciprofloxacin alone and in combination with diflunisal at each time point, as well as with the 0.5 μg/mL and 2 μg/mL treatments. However, there were statistically significant differences in *S. aureus* growth among the control group, the 0.5 μg/mL, and the 2 μg/mL ciprofloxacin concentrations, both individually and in combination with diflunisal.

**Figure 6:**
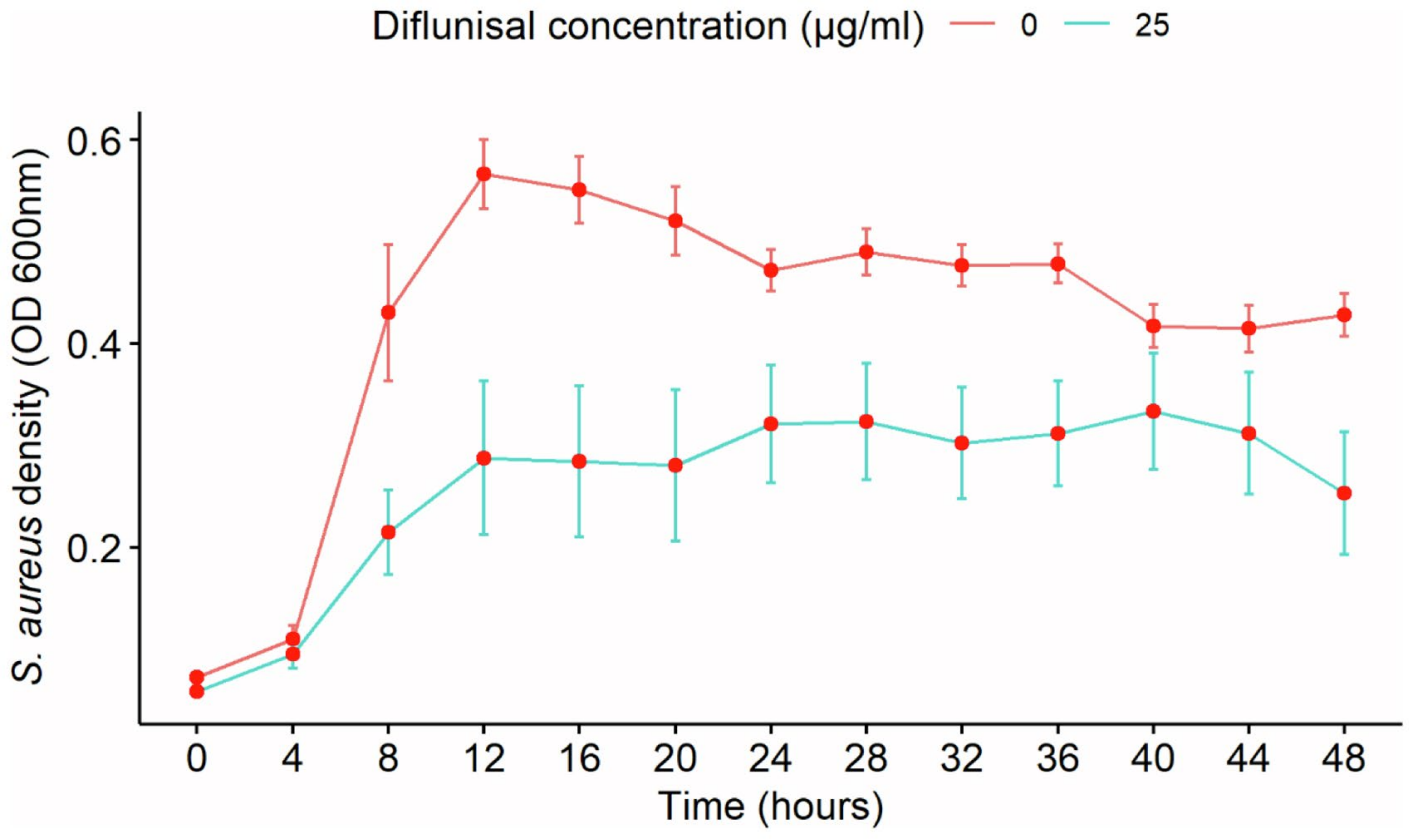
*Staphylococcus aureus* planktonic cell density over time, treated with diflunisal. The NSAID showed a bacteriostatic effect on planktonic cell growth from 12 to 48 h. Data are represented as the average of the *S. aureus* isolates planktonic cell density (OD 600 nm) ± standard error of mean (SEM) over time depending on the presence of diflunisal. The repeated measure ANOVA was significant at *p* = 0.00014.

The effect of time using diflunisal was evaluated individually against *S. aureus* growth and compared to bacterial growth without any treatment (control group). As shown in Figure 6, diflunisal exerted a bacteriostatic effect over *S. aureus* planktonic cells from 12 to 48 h. A two-way interaction was observed between diflunisal presence and time. In addition, there was a statistical difference regarding control over bacterial growth between the control group (no treatment) and diflunisal treatment from 12 to 20 h and 28 to 36 h of incubation.

### 3.6 *Staphylococcus aureus* biofilm viability after treatments

**Figure 7:**
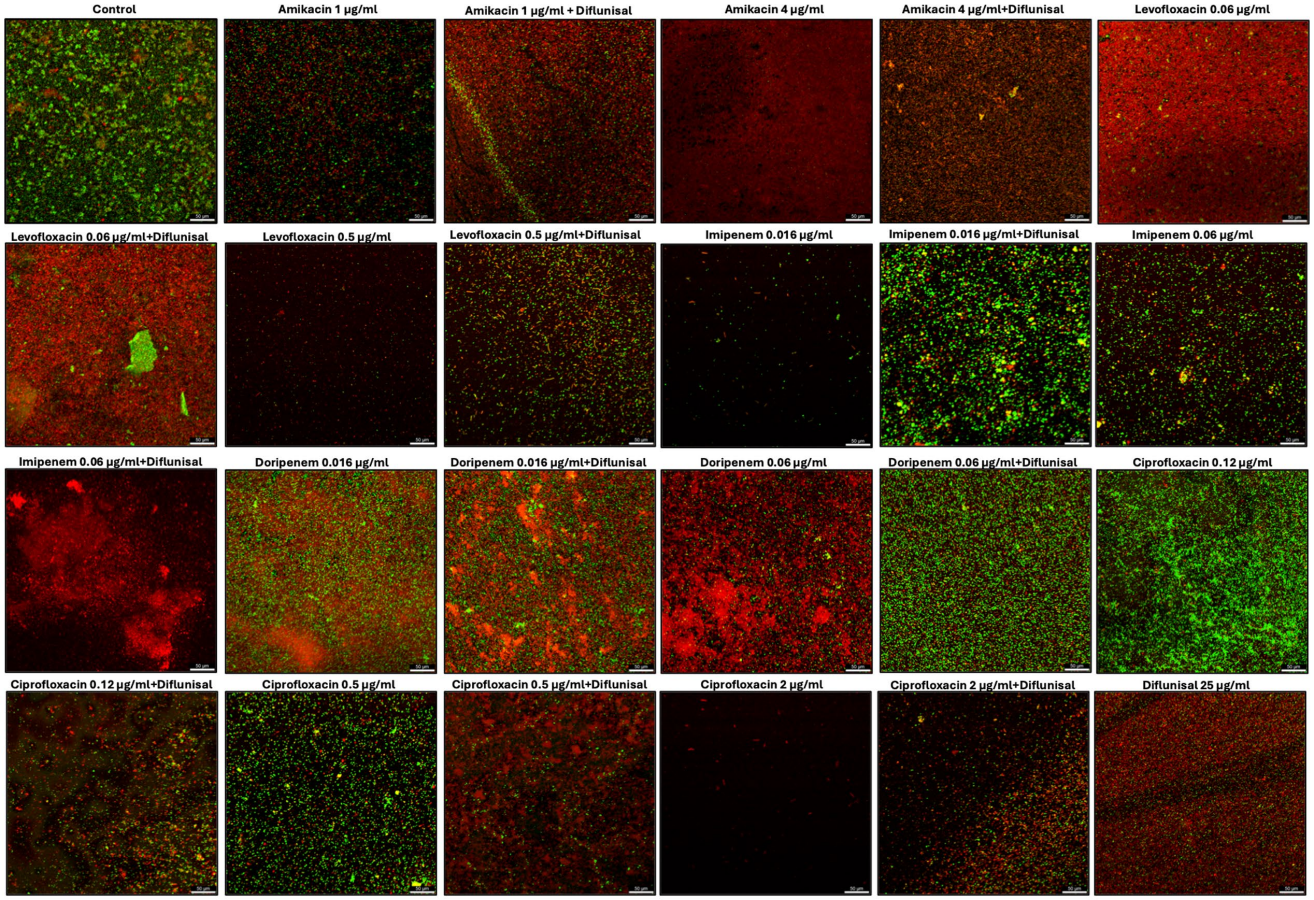
*Staphylococcus aureus* biofilms viability visualized by live/dead staining and CLSM. Biofilms were treated with antibiotics (amikacin, levofloxacin, imipenem, doripenem, and ciprofloxacin) alone and in combination with diflunisal for 48 h and stained with Syto 9 and Propidium iodide. The green color indicates live bacterial cells and red indicates dead bacterial cells.

Cell images captured using a live/dead assay with CLSM were able to show the effect of the treatments and cell viability within the biofilm matrix (Figure 7). The untreated biofilm (control) exhibited a high prevalence of live cells (green), indicating large viability in the absence of treatment. At 1 μg/mL of amikacin the image shows a mix of live and dead cells within the biofilm, suggesting a partial bactericidal effect at this concentration. When combined with diflunisal, the red staining increased (dead cells), which could indicate an enhanced killing activity. At 4 μg/mL, amikacin alone shows greater cell death, and this effect is maintained when combined with diflunisal.

For levofloxacin, the cells within the biofilm are mostly dead when treated with 0.06 μg/mL and when combined with diflunisal, the effect is sustained. When levofloxacin was used individually at 0.5 μg/mL there was a smaller number of aggregated cells, and most of them showed to be dead. However, when levofloxacin was combined with diflunisal, *S. aureus* viability increased, as well as cell aggregation.

Biofilms treated with 0.016 μg/mL of imipenem alone, showed few cells aggregation with a predominance of live cells. The addition of diflunisal showed an increase in dead and live cells, but also greater cell aggregation. At 0.06 μg/mL, imipenem alone had partial bactericidal effect, but when combined with diflunisal, there is a substantial increase in cell death.

Doripenem at 0.016 μg/mL alone and in combination with diflunisal showed limited effectiveness, with mostly live cells. At 0.06 μg/mL, doripenem became more effective in terms of bactericidal activity. However, when combined with diflunisal, bacterial cell viability in the biofilm increased.

With ciprofloxacin at 0.12 μg/mL, there was high cell aggregation and viability of live cells, suggesting poor effectiveness. However, when combined with diflunisal, the amount of dead cells increased. The biofilm treated with 0.5 μg/mL of ciprofloxacin alone showed moderate cell viability, but when combined with diflunisal, there was an increase in dead cells. At 2 μg/mL of ciprofloxacin used as an individual treatment, less cell aggregation and high cell death was observed. However, the combination of ciprofloxacin with diflunisal resulted in an increase in cell viability and density.

Regarding diflunisal individual treatment (at 25 μg/mL), it showed a greater cell aggregation when compared to antibiotics treatments. Nonetheless, there is a high prevalence of dead cells.

## 4 Discussion

This project explored an alternative to control *S. aureus* biofilm formation by testing various antibiotics considering concentrations and exposure time. Our guiding hypothesis was that combining antibiotic treatments with diflunisal would prevent biofilm formation, acting as a potential synergistic mechanism to enhance the treatments. Overall, this research found that treatment concentration and exposure time play a fundamental role in the effectiveness of the antibiotics, more so than the addition of the NSAID.

*Staphylococcus aureus* is one of the most common species found in chronic wound infections (Archer et al., 2011). In its biofilm state, it has been linked to life-threatening infections such as endocarditis, bloodstream infections caused by central lines, pneumonia caused by ventilators, implant-associated infections, and surgical site infections (Dauros-Singorenko et al., 2020). The biofilm matrix produced by *S. aureus* during the infective process increases resistance to antibiotics and affects the host immune response, resulting in recurring and untreatable illnesses (Pietrocola et al., 2022). NSAID are commonly used drugs prescribed to treat pain and control inflammation (Ghlichloo and Gerriets, 2022), and *S. aureus* has been observed to exhibit altered microbial and biofilm characteristics in the presence of these drugs.

The results showed that antibiotics alone limited *S. aureus* biofilm growth; however, biofilm formation was not completely inhibited. This aligns with a previous study showing that to inhibit biofilm formation, an increase in the antibiotic MIC is required with respect to was is recommended for planktonic cells (Bhattacharya et al., 2015). Microbial cells within a biofilm have been shown to be less susceptible to antibiotics compared to planktonic cells (Chen et al., 2020). Sub-MIC, which is a concentration lower than the MIC, may not be able to inhibit bacterial growth but can have other effects, such as altering gene expression and virulence factor production (Narimisa et al., 2020). Studies have demonstrated that the sub-MICs can enhance biofilm formation in some bacterial species, including *S. aureus*. In 2023, Azzam et al. reported that the sub-MIC of certain antibiotics, such as gentamicin, ceftriaxone, ampicillin, and norfloxacin, significantly induced biofilm formation in all *S. aureus* strains tested and found that the low concentrations induced the expression of *atl* and *sarA* genes which are involved on biofilm production (Azzam et al., 2023). Consistent with these findings, Lima-e-Silva et al., 2017 found that sub-MICs of rifampicin increase *S. aureus* biofilm formation. Low concentrations of antibiotics can create a stressful environment that stimulates biofilm formation as a mechanism of protection (Kaplan, 2011). However, in other studies, sub-MICs of antibiotics have been reported to reduce or inhibit biofilm formation on different bacterial species. Yang et al. (2017) demonstrated that five antibiotics significantly reduced *S. aureus* biofilm density. In our study, we were able to observe an increase in the biofilm density using lower concentrations of the antibiotics; however, it was not statistically significant, irrespective of diflunisal. This could be attributed to several factors, such as the type and concentration of the antibiotics used, the strains, the media used for bacterial and biofilm growth, and incubation time, among others. Moreover, we observed that diflunisal itself showed antimicrobial properties, which could counteract the potential enhancement effect on biofilm density by the low concentrations of antibiotics.

Biofilm growth is known to provide bacteria with higher protection against antibiotics, making them difficult to eradicate (Howlin et al., 2015). Thus, the biofilm matrix acts as a physical barrier that limits antibiotic penetration, which prevents the compound from reaching its target (Shin et al., 2021). Bacterial cells inside the biofilm matrix may enter a persistent state, which can also be a viable but non-culturable state (VBNC). Pasquaroli et al., 2013 reported that antibiotics could trigger VBNC in *S. aureus* biofilms. Since none of the treatments eradicated the biofilm in our study, it is possible that cells become VBNC under the stress of antimicrobials, resulting in low metabolic rates and cell proliferation, which also promotes tolerance to antibiotics, contributing to treatment failure (Archer et al., 2011; Peyrusson et al., 2022). However, the mechanisms by which cells become VBNC are not well elucidated, and it is believed that factors such as treatment type, exposure, strain, and others are influencing this state (Pasquaroli et al., 2014). Moreover, we also observed that the effect of the antibiotic concentration on biofilm density was time-dependent, which complies with Chen et al., 2020, where it was demonstrated that antibiotic concentration and extension of exposure time increased growth control over *S. aureus* biofilm, which suggests that extending the duration of antibiotic exposure potentially enables a complete biofilm eradication (Liu et al., 2021).

When treating *S. aureus* biofilms with antibiotics in combination with diflunisal, it was observed that at the lowest concentration, the NSAID produced a decrease (not statistically significant) in the efficacy of the treatment control in *S. aureus* biofilm growth. On the other hand, when using the highest concentration of the antibiotics, the effect of diflunisal in combination with the antibiotics was comparable to the outcome achieved with the antibiotics alone. However, there was no significant difference in the effect of biofilm density between the antibiotics alone and with diflunisal. These results differ from previous studies, which report that diflunisal blocks the accessory gene regulator system in *S. aureus* which could lead to the inhibition of virulence factors like biofilm formation expression (Yarwood and Schlievert, 2003; Khodaverdian et al., 2013; Spoonmore et al., 2020). However, Bernabè et al., 2021, demonstrated that a diflunisal analog (azan-7) enhanced MRSA susceptibility to clindamycin in planktonic and biofilm growth, but it did not show an anti-biofilm effect.

One of the key aspects of the CLSM images obtained after performing the live/dead assay was the ability to visually demonstrate what was expressed using OD values. For most antibiotics, diflunisal combinations caused an increase in dead cells, particularly at lower concentrations. However, in some cases, such as with levofloxacin at 0.5 μg/mL, imipenem at 0.016 μg/mL, doripenem at 0.06 μg/mL, and ciprofloxacin at 2 μg/mL, the combination of diflunisal led to increased biofilm viability. This suggests that while diflunisal may not enhance the antibiotics effect in reducing biofilm density, it could increment *S. aureus* cell death with the use of certain antibiotics, and its impact can be concentration dependent.

The ability of *S. aureus* to form biofilms is attributed to the utilization of various pathogenesis mechanisms and the quorum sensing mechanism mediated by the Agr system (Peng et al., 2022). The Agr system, composed of the accessory gene regulator (agr) locus, plays a crucial role in coordinating the expression of numerous virulence factors and biofilm-related genes (Zhou et al., 2020). Some studies have reported that diflunisal inhibits AgrA binding to the DNA sequence that corresponds to the promoter 3 (P3), which is required for RNA III, essential for cell communication, virulence factor expression, and biofilm formation (Reyes et al., 2011; Khodaverdian et al., 2013; Le and Otto, 2015; Chan et al., 2023). Our findings showed that diflunisal demonstrated antimicrobial and antibiofilm effects when used individually on *S. aureus,* which let us presume that it interfered with the Agr system. However, it is possible that blocking Agr does not stop *S. aureus* from producing a biofilm, and *S. aureus* could still utilize other mechanisms independent of the Agr system, as reported by Yarwood et al., 2004 and He et al., 2023.

Given the lack of significant differences observed between the impact of antibiotics used alone versus in combination with diflunisal on *S. aureus* biofilm formation, we sought to investigate whether this combination might cause a significant effect on controlling planktonic growth. Hence, we assessed the potential synergistic effect of antibiotics with diflunisal, specifically on *S. aureus* planktonic cells over time. The effect of antibiotic concentration on *S. aureus* planktonic growth was dose and time-dependent, as we found that higher doses of antibiotics, regardless of diflunisal status suppressed planktonic cell growth, and the effect of concentration depended on the time of exposure as well, which let us presume that the antibiotics had a hybrid bacteriostatic effect in the *S. aureus* isolates used in this study (Reed, 2000; Bernier and Surette, 2013). These antibiotics, commonly used in Gram-negative bacterial infections, exhibit potent activity against *S. aureus*, which has led to their exploration and proposed use as treatments for resistant strains (Ribes et al., 2010; Amin et al., 2015; Ghazi et al., 2017; Broussou et al., 2018). Furthermore, these antibiotics have been extensively studied in combination with other drugs to augment their antimicrobial efficacy. A study demonstrated that levofloxacin, in combination with acrylic bone cement and lactose, exhibits antimicrobial effects against *S. aureus* planktonic and biofilm growth (Ferreira et al., 2017) and ciprofloxacin activity has been enhanced using a combination with other antimicrobials against *S. aureus* planktonic growth (Yasir et al., 2021). However, in this study, there was no enhancement in the bacteriostatic effect of the target antibiotics by diflunisal. Overall, our findings suggest that the interaction between diflunisal and antibiotics depends on the specific antibiotic, its concentration, and the exposure time. Diflunisal’s impact varies from diminishing the efficacy of certain antibiotics (amikacin at lower concentrations) to enhancing the control of planktonic growth in other cases (doripenem at lower concentrations). The effect of the antibiotics alone and combined with diflunisal on *S. aureus* planktonic growth were not statistically significant, suggesting that the combination of the target antibiotics and NSAID may not be an effective strategy to prevent *S. aureus* infections. However, diflunisal used individually poses antimicrobial properties against *S. aureus* biofilm and planktonic cell growth.

These findings highlight the importance of a rigorous compound selection for combination therapy strategies in clinical settings, and further studies must evaluate the effect of NSAIDs combined with antibiotics on pre-formed mature biofilms to identify if these are useful for biofilm prevention, eradication, or both.

## 5 Study limitations

Future investigations should include large-volume biofilm models to evaluate the effect of the selected antibiotic-diflunisal combinations under real-world scenarios. Additionally, future experiments should compare biofilm density (OD 580 nm) and cell density (OD 600 nm) in planktonic growth with CFU counts after treatments. This approach would provide a more comprehensive understanding of the relationship between biofilm density and viable cell counts, offering broader insights into the effectiveness of the treatments.

On the other hand, our current study focused on pre-treatment to observe its effects in biofilm formation. However, investigating these combinations as an intervention to reduce existing biofilms would provide valuable information on their potential as therapeutic agents in established infections.

Finally, the limited range of antibiotic concentrations tested, and the use of 6 isolates under *in vitro* conditions may have restricted the applicability of the findings. Future studies should consider a broader range of antibiotic concentrations, bacterial strains, as well as exploring the molecular mechanisms underlying the treatment effects, to build a more comprehensive understanding of the interactions between NSAIDs and antibiotics in biofilm control.

## Data Availability

The original contributions presented in the study are included in the article/supplementary material, further inquiries can be directed to the corresponding author.
